# Dr. George Lattin

**Published:** 1900-05

**Authors:** 


DR. GEORGE LATTIN, of Cattaraugus, N. Y., died at his home on Wednesday, April 11, 1900. His funeral was largely attended the following Friday at 1.00 p.m. by his friends, neighbors and professional colleagues.

